# Repression of exogenous gene expression by the retinoic acid target gene G0S2

**DOI:** 10.3892/ijo.2013.1876

**Published:** 2013-03-28

**Authors:** TIAN MA, JESSICA P. DONG, DAVID J. SEKULA, DENNIS LIANG FEI, WILLIAM W. LAMPH, MICHAEL HENDERSON, YUN LU, STEVEN BLUMEN, SARAH J. FREEMANTLE, ETHAN DMITROVSKY

**Affiliations:** 1 Departments of Pharmacology and Toxicology, Geisel School of Medicine at Dartmouth, Hanover, NH 03755; 2 Medicine, Geisel School of Medicine at Dartmouth, Hanover, NH 03755;; 3 Norris Cotton Cancer Center, Dartmouth-Hitchcock Medical Center, Lebanon, NH 03756;; 4 Dartmouth College, Hanover, NH 03755;; 5 Ligand Pharmaceutical, San Diego, CA 92121, USA

**Keywords:** retinoic acid, G0/G1 switch gene 2, transcriptional repression

## Abstract

The G0/G1 switch gene 2 (G0S2) is rapidly induced by all-*trans*-retinoic acid (RA)-treatment of acute promyelocytic leukemia (APL) and other cells. G0S2 regulates lipolysis via inhibition of adipose triglyceride lipase (ATGL). This study found that retinoic acid receptor (RAR), but not retinoid X receptor (RXR) agonists induced G0S2 expression in APL cells. Novel G0S2 functions were uncovered that included repression of exogenous gene expression and transcriptional activity. Transient G0S2 transfection repressed the activities of multiple reporter constructs (including the retinoid-regulated species RARβ, UBE1L and G0S2); this occurred in diverse cell contexts. This inhibition was antagonized by siRNA-mediated G0S2 knockdown. To determine the inhibitory effects were not due to transient G0S2 expression, G0S2 was stably overex-pressed in cells without appreciable basal G0S2 expression. As expected, this repressed transcriptional activities. Intriguingly, transfection of G0S2 did not affect endogenous RARβ, UBE1L or G0S2 expression. Hence, only exogenously expressed genes were affected by G0S2. The domain responsible for this repression was localized to the G0S2 hydrophobic domain (HD). This was the same region responsible for the ability of G0S2 to inhibit ATGL activity. Whether an interaction with ATGL accounted for this new G0S2 activity was studied. Mimicking the inhibition of ATGL by oleic acid treatment that increased lipid droplet size or ATGL siRNA knockdown did not recapitulate G0S2 repressive effects. Engineered gain of ATGL expression did not rescue G0S2 transcriptional repression either. Thus, transcriptional repression by G0S2 did not depend on the ability of G0S2 to inhibit ATGL. Subcellular localization studies revealed that endogenous and exogenously-expressed G0S2 proteins were localized to the cytoplasm, particularly in the perinuclear region. Expression of a mutant G0S2 species that lacked the HD domain altered cytosolic G0S2 localization. This linked G0S2 subcellular localization to G0S2 transcriptional repression. The potential mechanisms responsible for this G0S2 repression are examined.

## Introduction

All-*trans* retinoic acid (RA) is a derivative of vitamin A, which is required for development, vision and immune function (1–3). As a signaling molecule, RA affects target gene transcription through retinoid receptor-mediated mechanisms (4). There are two families of nuclear retinoid receptors: retinoic acid receptors (RARs) and retinoid X receptors (RXRs). RXR/RAR heterodimers and RXR homodimers exist; these respective complexes bind to defined retinoic acid response elements (RAREs) in the promoter regions of retinoid target genes, as reviewed (4). In the absence of RA-treatment, these receptors basally associate with an inhibitory co-repressor complex and upon RA-treatment a stimulatory co-activator complex is recruited that leads to chromatin remodeling and retinoid target gene transcription, as reviewed (4).

In addition to its physiological roles, RA is also used as therapy for acute promyelocytic leukemia (APL) (4,5). RA-treatment of APL is a successful example of differentiation therapy. The majority of clinical APL cases exhibit a balanced chromosomal translocation t(15;17), resulting in a fusion protein between the promyelocytic leukemia (PML) and the retinoic acid receptor-α (RARα) gene products (6,7). This fusion protein retains the ability to bind to an RARE, but also has a strong association with its co-repressor complex, as reviewed (7). Physiological retinoid levels are not able to dissociate the co-repressor complex, resulting in transcriptional repression of retinoid target genes. Since these target genes are critical for induced cellular differentiation, basal repression of transcription of these species can block maturation of immature promyelocytes, leading to APL (7). In contrast, pharmacological concentrations of RA can overcome the inhibitory association between the co-repressor complex and the PML/RARα fusion protein and can recruit a stimulatory co-activator complex that leads to the transcription of retinoid target genes. One consequence is retinoid-induced degradation of the PML/RARα fusion protein, as reviewed (8). Together, these pathways contribute to the maturation of APL cells and clinical remission of APL patients.

Clinical use of retinoids is limited by toxicity and resistance (4,7). In a search for retinoid target genes that could serve as candidate therapeutic targets in APL, the G0/G1 switch gene 2 (G0S2) was found. G0S2 is one of the most rapid and prominently-induced RA target genes in APL (9,10). G0S2 is a small basic protein with 103 amino acids (11). It does not have apparent homology to other proteins and its functions are under intensive study. The G0S2 gene was discovered in a screen to identify species regulated in the lectin-induced G0 to G1 cell cycle change of human peripheral blood mononuclear cells (11). However, its precise role in cell cycle regulation has been elusive (11). G0S2 is expressed in white and brown adipose tissue; it is highly expressed in the liver, heart and skeletal muscle (12,13).

G0S2 is a regulator of lipolysis (13). It is a target of the peroxisome-proliferator-activated receptor γ (PPARγ) in adipocytes and G0S2 is upregulated in adipogenesis (12). G0S2 is also known to regulate adipose lipolysis through its inhibition of adipose triglyceride lipase (ATGL) activity (13). In settings of high metabolic demand, ATGL mediates hydrolysis of triglyceride (TAG) stored in lipid droplets of adipocytes to diglyceride (DAG) and free fatty acid (FFA) for subsequent energy use. It is through its hydrophobic domain (HD) that G0S2 binds to ATGL, which can inhibit lipolysis (13). As expected, G0S2 knockdown was found to enhance lipolysis in adipocytes, whereas G0S2 overexpression reduced lipolysis; this resulted in TAG accumulation and an increase in lipid droplet size (13).

G0S2 is involved in diverse cellular activities. For example, G0S2 is upregulated after treatment with the lymphocyte mitogen lectin and downregulated in peripheral blood mono-nuclear cells by treatment with the immunosuppressive agent cyclosporine (11,14). G0S2 is also upregulated in peripheral blood or bone marrow-derived mononuclear cells isolated from patients with different autoimmune diseases, including psoriasis, rheumatoid arthritis, vasculitis and lupus (15–17). Although engineered G0S2 transgenic mice did not exhibit evidence for an autoimmune disease, these mice did have autoimmunity-related antibodies in their serum (17). Together, these findings implicated a role for G0S2 in immune regulation.

G0S2 was proposed to act as a tumor suppressor. This hypothesis came about from evidence for hypermethylation of the G0S2 promoter that conferred its silencing in head and neck squamous cell carcinomas (18,19) and squamous cell lung carcinomas (20,21). G0S2 overexpression also augmented apoptosis in lung and colon cancer cells by interacting with Bcl-2, which in turn antagonized the formation of anti-apoptotic Bcl-2/Bax heterodimers (22). These studies were consistent with a tumor suppressive role for G0S2.

In this study, G0S2 was shown to be induced in APL cells after treatment with RAR, but not RXR agonists. A previously unrecognized function of G0S2 was uncovered. G0S2 was found to repress exogenous gene expression and reporter activity. Yet, G0S2 did not affect endogenous expression of the examined species. These inhibitory effects were not restricted to APL cells, but were also detected in diverse cellular contexts, including those that were retinoid differentiation-responsive or not. The studies reported here indicate that these inhibitory G0S2 effects were mediated through an overlapping domain that conferred ATGL repression and altered G0S2 subcellular localization. Yet, this new G0S2 function was not rescued by gain of ATGL expression or mimicked by antagonizing ATGL activity. Thus, these findings revealed that these G0S2 effects are independent of its previously recognized role in regulating ATGL activity. The biological implications of this G0S2 repression are discussed.

## Materials and methods

### Cell culture and reagents

Cell lines were cultured in their respective media supplemented with penicillin (100 U/ml) and streptomycin (100 *μ*g/ml) (Mediatech, Manassas, VA) in a humidified incubator at 37°C with 5% CO_2_. The NB4 human APL cell line (9) was cultured in advanced RPMI-1640 media supplemented with 2% fetal bovine serum (FBS) and 4 mM L-glutamine. BEAS-2B immortalized human bronchial epithelial cells were cultured in LHC-9 media, as before (23). The human 293T embryonic kidney cell line (ATCC, Manassas, VA) was cultured in DMEM media supplemented with 10% FBS. The multipotent NTERA-2 clone D1 (NT2/D1) human embryonal carcinoma cells were cultured in DMEM media supplemented with 10% FBS and 2 mM L-glutamine (24). Murine ED-1 (25) and the human A549 (ATCC) lung cancer cell lines were each cultured in RPMI-1640 media supplemented with 10% FBS. To engineer cell lines with stable G0S2 expression, ED-1 cells were transduced with a G0S2 lentivirus (Addgene, Cambridge, MA) (designated as ED-1-G0S2) and comparisons were made to an insertless control lentivirus (ED-1-vector). Cells were then selected in media supplemented with blasticidin S HCl (17.43 *μ*M, Invitrogen, Grand Island, NY) in RPMI-1640 media supplemented with 10% FBS.

### Independent retinoid and rexinoid effects on G0S2 expression

NB4 APL cells were individually treated for 2 days with the RAR (RA, 1 *μ*M) or RXR (LG268, 1 *μ*M; Ligand Pharmaceutical, La Jolla, CA) agonists. The proteasome inhibitors MG132 (Calbiochem) and ALLN (Calbiochem), protease inhibitors PMSF (Sigma, 1 mM) and EDTA (Sigma, 1 mM) and the lysosomal inhibitor NH4Cl (Sigma, 2 mM) were each purchased. Five hours after transfection, the original transfection medium was removed and replenished with fresh media supplemented individually with each of these inhibitors, except for the protea-some inhibitors (MG132, 10 *μ*M and ALLN, 50 *μ*M), which were each added 44 h after transfection. Luciferase assays were performed 48 h after transfection.

### Plasmids and siRNAs

For luciferase assay experiments, pRL-TK (Promega, Madison, WI), pGL3E (Promega), βRARE-TK-luc (26), pGL3-UBE1L-TK-luc (27) and pGL3-G0S2-FL-luc (9) were respectively used as reporter constructs. For G0S2 gain of expression experiments with the CMV-myc-G0S2 (myc-G0S2) vector (9), the results were compared to its empty vector (CMV-myc ΔHD) as a control (9). The ΔHD G0S2 mutation of CMV-myc-G0S2 [myc-G0S2 was generated by polymerase chain reaction (PCR) assays with deletions accomplished using primers that flanked the region to be deleted in the full length CMV-myc-G0S2 vector. Primer sequences were: forward primer 5′-GATGGTGAAGCTGATGGAGACTGTGTGCAGC-3′ and reverse primer 5′-CACAGTCTCCATCAGCTTCACCATCTTCCC-3′. For ATGL engineered overexpression experiments, the pCMV-SPORT6-ATGL vector (Thermo Fisher, Rockford, IL) was used to overexpress murine ATGL. An empty vector pCMV-SPORT6 served as a control vector. Target sequence for G0S2 siRNA (Thermo Fisher) was: 5′-AGATGGTGAAGCTGTACGT-3′. The target sequences for ATGL siRNAs (Thermo Fisher) were: human ATGL siRNA1: 5′-GTAAAGATCATCCGCAGTT-3′ and human ATGL siRNA2: 5′-GGGCGAGAGTGACATCTGT-3′; and for murine ATGL siRNA1: 5′-GAAATTGG GTGACCATCTG-3′; and murine ATGL siRNA2: 5′-GGAGAGAACGTCATCATAT-3′. A non-targeting RISC-free siRNA (Thermo Fisher) was used as a control.

### Transient transfection and luciferase assays

NB4 APL cells were transiently co-transfected with myc-G0S2 or a corresponding empty vector control with the indicated luciferase construct using the AMAXA cell line Nucleofector kit V (Lonza, Basel, Switzerland) according to the manufacturer’s protocol. Following transfection, cells were plated at 2×10^6^ cells/ml in individual wells of a 12-well tissue culture plate and treated with RA (1 *μ*M) or dimethyl sulfoxide (DMSO) as vehicle control for 6 h. Cells were then harvested in Passive Lysis Buffer as part of the Dual-Luciferase Reporter Assay System kit (Promega). Analyses for luciferase activities were performed according to the manufacturer’s recommended protocol and luciferase activity was measured with a TD-20/20 Luminometer (Promega). Renilla luciferase activity was also measured. To normalize for total protein, total protein concentrations within studied cell lysates were measured using the BCA protein assay kit (Thermo Fisher). Luciferase activities were normalized to the respective cellular protein concentrations and activities were subsequently normalized to the vehicle-treated insertless vector experimental arm. Similar transfection efficiencies were confirmed by co-transfecting fluorescein-labeled siRNA or GFP in desired cells and then by measuring fluorescein or GFP-positive cells using flow cytometry (Becton Dickinson FACScan cytometer, Franklin Lakes, NJ or MACSQuant VYB, Miltenyi Biotec, Bergisch Gladbach, Germany). Cell lysates were harvested in radioimmunoprecipitation assay (RIPA) buffer (Thermo Fisher) supplemented with protease arrest (GBioscience, St. Louis, MO) for immunoblot analysis to confirm that G0S2 knockdown or engineered overexpression was achieved in the desired cells.

BEAS-2B, NT2/D1, ED-1, A549, ED-1-G0S2 and ED-1-vector cells were individually plated at densities of 2×10^5^, 2×10^5^, 3.5×10^4^ to 1×10^5^, 6×10^5^, 2×10^5^ and 2×10^5^ cells/well in 6-well tissue culture plates, respectively. BEAS-2B cells were transiently transfected the next day with indicated constructs using Fugene 6 (Roche, Indianapolis, IN). ED-1, NT2/D1 and A549 cells were individually transfected with Lipofectamine 2000 (Invitrogen). ED-1-G0S2 and ED-1-vector cells were each transfected with TransIT-LT1 transfection reagent (Mirus, Madison, WI) using the respective manufacturer’s protocol. Twenty-four hours after transient transfection, the medium was replaced with fresh medium supplemented respectively with RA or DMSO as a vehicle for NT2/D1 and BEAS-2B cells, and with fresh media for ED-1 and A549 cells. For oleic acid treatment experiments, varying concentrations of oleic acid (Sigma, St. Louis, MO) were added 24 h after transfection; cell lysates for the NT2/D1, BEAS-2B, ED-1 and A549 cell lines were individually harvested 48 h after transfection for luciferase activity analyses. Cell lysates for stably transfected ED-1-G0S2 and ED-1-vector cells were harvested 24 h after transfection to measure luciferase activity and also placed in RIPA buffer for immunoblot analyses.

### Real-time PCR assays

To evaluate effects of G0S2 transient transfection on endogenous gene expression, ED-1 cells were transfected with the myc-G0S2 vector. RA or vehicle (DMSO) was added 24 h after transfection. Total RNA was isolated 48 h after transfection using TRIzol reagent (Invitrogen). Reverse transcription was performed using the High Capacity cDNA Reverse Transcription Kit (Life Technologies, Carlsbad, CA) with a Peltier Thermal Cycler (GMI, Ramsey, MN). Real-time PCR assays were performed using SYBR-Green PCR master mix (Life Technology) with the 7500 fast Real-time PCR system (Life Technology). Primer sequences were as follows: murine RARβ forward primer: 5′-CAGTGAGCTGGCCACCAAGT-3′; reverse primer: 5′-GCGATGGTCAGACCTGTGAA-3′; murine UBE1L forward primer: 5′-CTACGAGCGACTCCATATACCT-3′; reverse primer: 5′-TACACACAGGGTAGGGAGCAT-3′; murine G0S2 forward primer: 5′-AGTGCTGCCTCTCTTCCCAC-3′; reverse primer: 5′-TTTCCATCTGAGCTCTGGGC-3′; murine GAPDH forward primer: 5′-AGGTCGGTGTGAACGGATTTG-3′ and reverse primer: 5′-TGTAGACCATGTAGTTGAGGTCA-3′.

### Subcellular localization and immunoblot analysis

For subcellular localization of endogenous G0S2, NB4 cells were plated at 105/ml and treated with RA (1 *μ*M) for 48 h. Cells were then harvested and fractionated using the Subcellular Protein Fractionation Kit according to manufacturer’s protocol (Thermo Fisher, Rockford, IL). To confirm that the ΔHD G0S2 mutant protein was of the expected size and to establish that respective gain or loss of G0S2 or ATGL expression was achieved, myc-G0S2 and myc-G0S2 ΔHD plasmids were individually transfected into 293T cells. Human ATGL siRNAs were individually transfected into A549 cells, and murine ATGL siRNA and the pCMV-SPORT6-ATGL vector were each transfected into ED-1 cells. Cell lysates were harvested 48 h after transfection in RIPA buffer (Thermo Fisher) with protease arrest (GBioscience) added. Protein concentrations were determined using the BCA Protein Assay Kit (Thermo Fisher). Samples were run on SDS-PAGE gels and transferred to nitrocellulose membrane, as before (9). Membranes were individually probed with antibody recognizing G0S2 (9) to detect endogenous or stably overexpressed G0S2 proteins with antibody recognizing myc (Covance, Princeton, NJ) to individually detect myc-G0S2 and myc-G0S2 ΔHD, with antibody recognizing ATGL (Cell Signaling, Danvers, MA) to detect both human and mouse ATGL species, with antibody recognizing transglutaminase II (TGase II) (Thermo Fisher), or with respective antibodies that recognized UBE1L (9) or RARβ (Santa Cruz Biotechnology, Santa Cruz, CA) proteins. Antibodies that recognized actin, COX-4 or nucleoporin (all from Santa Cruz Biotechnology) were used to confirm similar protein loadings were achieved for the desired subcellular immunoblot analyses.

### Confocal microscopy

ED-1 cells were plated at a density of 3×10^4^ cells/well on a poly-D-lysine (Sigma)-coated cover slip in individual wells of a 12-well tissue culture plate. Cells were transfected on the following day with the desired myc-tagged G0S2 expression or control constructs using Lipofectamine 2000 (Invitrogen) according to the manufacturer’s protocol. Twenty-four hours later, cells were fixed in 4% paraformaldehyde for 15 min, washed with phosphate-buffered saline (PBS), incubated in 0.1% Triton in PBS (PBT) for 10 min and blocked with 7.5% bovine serum albumin (BSA) in PBS overnight at 4°C. Cells were subsequently washed in 0.1% PBT solution and stained with an anti-myc antibody (Convance, at 1:100 dilution) in 1% BSA in PBT for 45 min at room temperature and with goat anti-mouse secondary antibody conjugated to Alexa Fluor 647 fluorophor (Invitrogen, at 1:1,000 dilution) in 1% BSA in PBT for 30 min at room temperature. F-actin was stained using Alexa Fluor 568 Phalloidin (Invitrogen, 1:40 dilution) in 1% BSA in PBT for 20 min at room temperature in the dark. Coverslips were gently washed with PBS three times before mounting onto slides using Prolong Gold with DAPI (Invitrogen) staining. The slides were viewed using a Zeiss LSM 510 confocal microscope (Zeiss, Oberkochem, Germany) and representative images were obtained.

### Statistical analysis

Two-sample t-tests were used for statistical analyses using Microsoft Excel software, with significance defined as a two-sided P<0.05.

## Results

### G0S2 inhibits exogenous reporter activity of several retinoid-regulated genes

**Figure 1:**
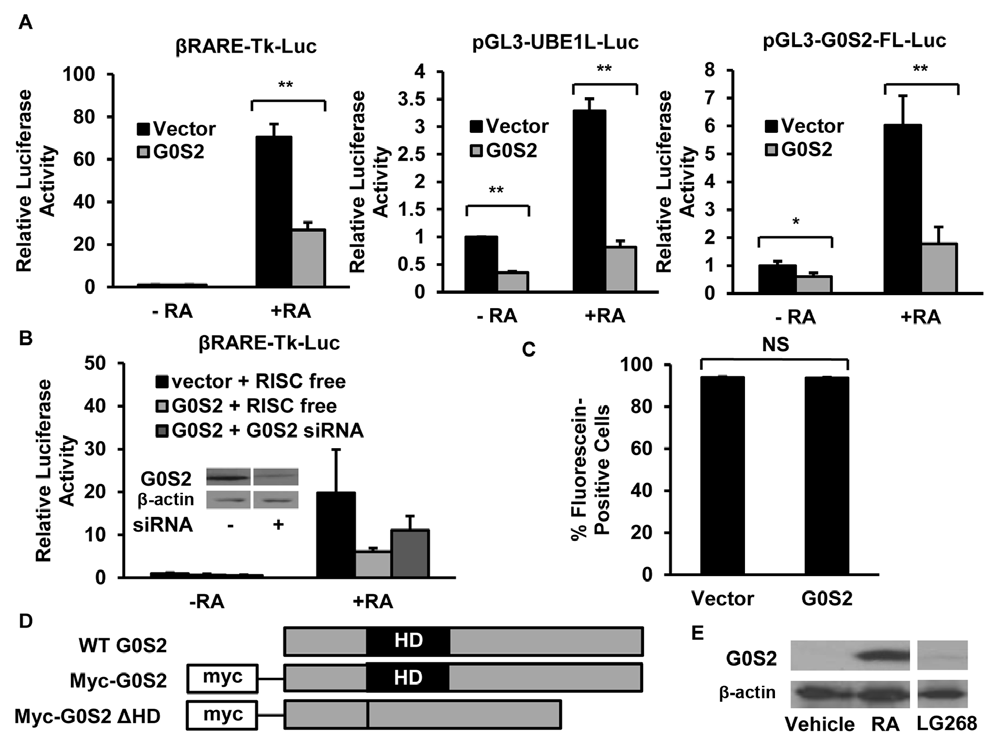
Transient G0S2 transfection reduced activity of different reporter constructs containing a retinoid responsive elemener constructs containing a retinoid responsive element. (A) G0S2 transient transfection independently decreased reporter plasmid activity of βRARE-Tk-Luc, pGL3-UBE1L-Luc or pGL3-G0S2-FL-Luc reporter constructs in NB4 APL cells, both in the presence and absence of RA (1 *μ*M)-treatment. (B) G0S2 knockdown was achieved using an siRNA that repressed G0S2 mRNA expression relative to a control siRNA. G0S2 knockdown partially rescued this transcriptional repression by G0S2 transfection. Immunoblot analyses confirmed the expected decline of G0S2 protein in these APL cells, as shown in the insert. (C) G0S2 transient transfection did not affect transfection efficiency of NB4 cells as compared to vehicle control, as indicated by scoring the percentage of cells transfected with fluorescein-linked siRNA by flow analysis. (D) Schematic of studied G0S2 constructs that respectively included wild-type (WT) G0S2, myc-tagged wild-type G0S2 (Myc-G0S2), and myc-tagged G0S2 with the hydrophobic domain (HD) deleted (myc-G0S2 ΔHD). (E) The immunoblot displayed in this panel indicates relative to vehicle (DMSO) control that G0S2 protein is induced by the RAR agonist RA (1 *μ*M), but not by the RXR agonist LG268 (1 *μ*M) in NB4 APL cells after 2 days of treatment. Representative results are shown from three independent experiments (each performed in triplicate) with error bars representing standard deviations. ^*^P<0.05 and ^**^P<0.01 depict statistical significance. NS indicates a comparison that is not significant.

Our prior study identified G0S2 as a direct retinoid target gene that was markedly induced after *in vitro* RA-treatment of cultured NB4 APL cells and leukemic cells from APL patients as well as after *in vivo* RA-treatment of transgenic APL mice (9). The current study confirmed and extended that prior work by showing that RAR but not RXR agonists induced G0S2 expression in NB4 APL cells (Fig. 1E).

**Figure 2:**
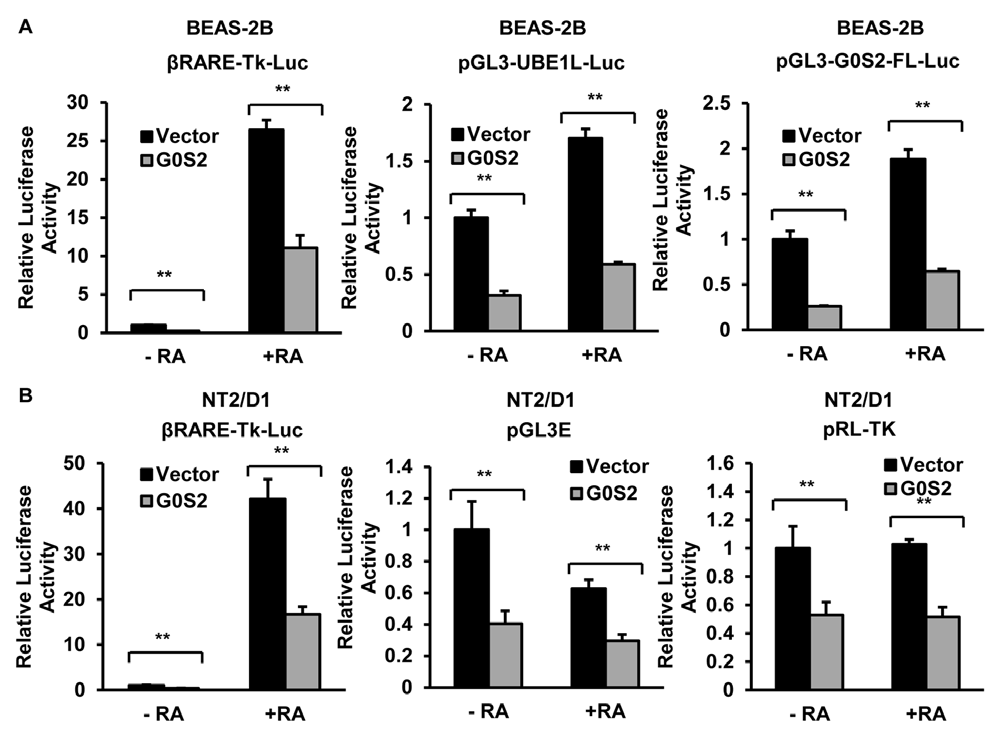
G0S2 inhibitory effects of G0S2 in retinoid differentiation or growth responsive cell contexts. (A) Transient transfection of G0S2 inhibited luciferase activity of retinoid responsive reporter plasmids βRARE-Tk-Luc, pGL3-UBE1L-Luc or pGL3-G0S2-FL-Luc in BEAS-2B cells, both in the presence and absence of RA (1 *μ*M) treatment. (B) Individual transient transfections in NT2/D1 cells (+/− RA-treatment, 1 *μ*M) of G0S2 inhibited activity of the reporter plasmid βRARE-Tk-Luc, which contained a retinoid responsive element, as well as the reporter plasmids pGL3E (firefly) and pRL-TK (renilla) that did not contain this responsive element. Representative results are shown from three independent experiments (each performed in triplicate) with error bars representing standard deviations. ^**^P<0.01 depict statistical significance.

To investigate the role of G0S2 in retinoid-dependent pathways, myc-tagged G0S2 was transiently transfected into NB4 APL cells along with the respective luciferase reporter constructs of the individual retinoid-regulated species: RARβ, UBE1L or G0S2 itself. Notably, exogenous G0S2 expression significantly (P<0.05) reduced individual reporter activity in NB4 APL cells for each of the respective reporter constructs (Fig. 1A). This repression was observed in the presence and absence of RA-treatment (Fig. 1A). As expected, G0S2 knockdown by an siRNA that targeted G0S2 for repression partially reversed these inhibitory effects versus a control siRNA (Fig. 1B). This decline in luciferase activity in NB4 cells was not due to changes in transfection efficiency, as shown in Fig. 1C. Similar inhibitory effects of G0S2 were observed in other retinoid-responsive cell lines including BEAS-2B immortalized human bronchial epithelial (23) and NT2/D1 multipotent human embryonal carcinoma (24) cell lines (Fig. 2). This inhibition by G0S2 was extended to include other reporter constructs that did not contain retinoid responsive elements such as pGL3E (firefly), which was the control vector for the indicated luciferase reporter constructs, and pRL-TK (renilla), as shown in Fig. 2B.

### The G0S2 hydrophobic domain (HD) mediates the G0S2 inhibitory effects

**Figure 3:**
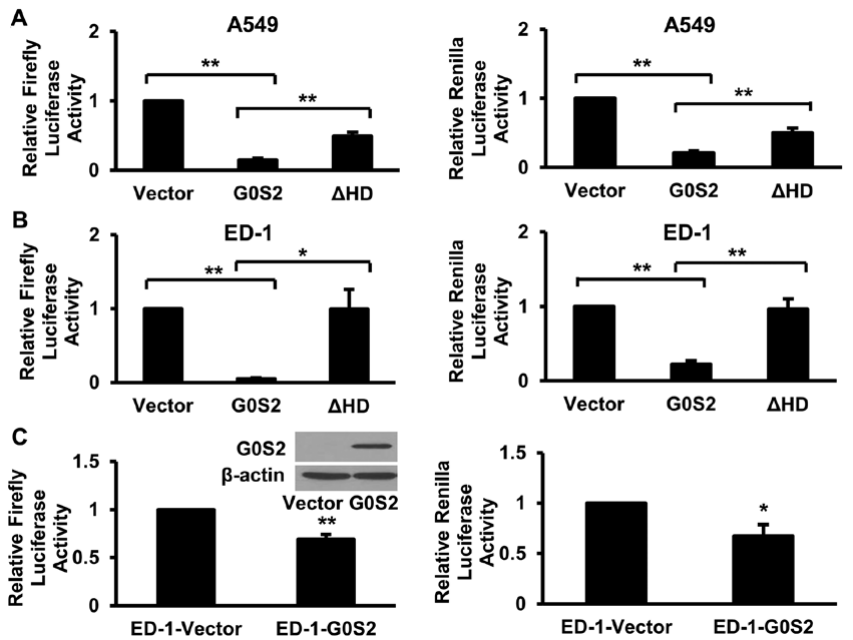
Inhibitory effects of G0S2 domains in different cell contexts. Transient transfection of G0S2 decreased the luciferase activity of the co-transfected PGL3E (firefly, left panel) and pRL-TK (renilla, right panel) reporter plasmids in (A) A549 cells and independently in (B) ED-1 cells. Transient transfection of G0S2 that lacked the HD domain (ΔHD) exhibited significantly less (P<0.01) repressive effects on luciferase activity than wild-type G0S2. (C) Stable overexpression of G0S2 in ED-1 cells also decreased the luciferase activity of the transiently transfected firefly (left panel) with insert displayed immunoblot confirmation of G0S2 overexpression relative to the insertless vector control transfectant) or renilla (right panel) reporter constructs. Results were shown from three independent experiments with error bars representing standard deviation. ^*^P<0.05 and ^**^P<0.01 represent statistical significance.

G0S2 was previously shown to require its HD domain (Fig. 1D) to inhibit ATGL activity (13). To explore whether this domain was also important for G0S2 inhibitory effects, myc-G0S2 ΔHD was individually co-transfected into ED-1 and A549 cells with firefly or renilla luciferase constructs. The G0S2 mutation designated as ΔHD in which the HD domain was removed had significantly (P<0.05) less ability to repress either firefly or renilla luciferase reporter activities than the full-length G0S2 species (Fig. 3A and B). To exclude the possibility that removal of the HD domain destabilized G0S2, immunoblot analysis was performed. The stability of this mutant G0S2 protein was similar to that of wild-type G0S2 (data not shown). Thus, the HD domain of G0S2 exerted this inhibitory effect of G0S2.

### Stable G0S2 expression inhibits reporter plasmid activity

To exclude the possibility that the G0S2 inhibitory effects were due to transient G0S2 co-transfection, G0S2 was stably expressed in ED-1 cells (ED-1-G0S2) in Fig. 3C. The consequences of this on luciferase reporter activities were next examined. As compared with ED-1 cells that were stably transfected with an insertless vector (designated as ED-1-vector cells), the G0S2 transductants significantly (P<0.05) inhibited firefly or renilla luciferase reporter activities (Fig. 3C). To exclude the possibility that G0S2 transfection activated protein degradation programs that would destabilize genetically-introduced proteins, transient transfections of luciferase reporter plasmids were repeated in 293T cells in the presence of transfected G0S2 and co-treatment with individual proteasomal, protease, or lysosomal inhibitors. These inhibitors did not abrogate the repressive effects of G0S2 expression on transcriptional activity (data not shown). Thus, induced protein destabilization did not appear to confer these inhibitory effects of expressed G0S2.

### G0S2 does not affect endogenous gene expression

**Figure 4:**
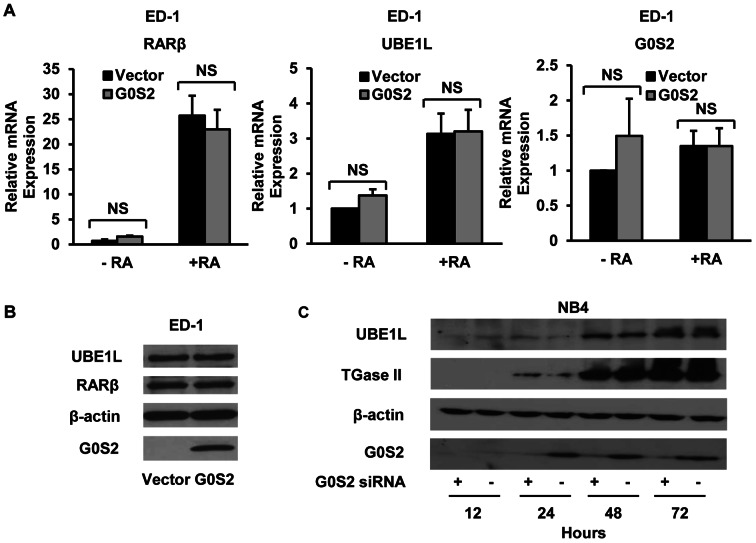
G0S2 does not affect endogenous retinoid target gene expression. (A) Transient transfection of G0S2 in to ED-1 cells did not affect the mRNA level of endogenous retinoid target genes RARβ, UBE1L or G0S2. Standard error bars are shown and results were averaged from three independent experiments. (B) Stable G0S2 overexpression achieved in ED-1 cells did not affect the endogenous protein levels of RARβ and UBE1L as compared to control ED-1 cells. (C) Knocking down endogenous G0S2 with siRNA also did not affect endogenous UBE1L and TGase II levels in NB4 cells after RA (1 *μ*M)-treatment. Actin served as a loading control. Three independent experiments were performed. NS indicates a comparison that is not significant.

To investigate G0S2 effects on endogenous expression of retinoid regulated species, G0S2 was transiently transfected into ED-1 murine lung cancer cells. The expression of endogenous RA-induced species was next examined after G0S2 transfection. In contrast to the marked ability of G0S2 to inhibit the activity of the individually co-transfected retinoid regulated RARβ, UBE1L and G0S2 reporter plasmids, transiently transfected G0S2 did not appreciably affect endogenous mRNA levels of these respective species, as displayed in Fig. 4A. Similarly, stable G0S2 overexpression in ED-1-G0S2 cells did not affect endogenous RARβ or UBE1L protein expression as compared to its control ED-1-vector transfected cells (Fig. 4B). In addition, siRNA-mediated knockdown of the endogenous G0S2 expression (induced by RA-treatment) in NB4 APL cells did not affect endogenous protein expression of the retinoid regulated genes UBE1L or TGase II (Fig. 4C). Together, these studies extended prior findings that G0S2 inhibited exogenously introduced reporter activities by showing that engineered gain or loss of G0S2 also did not affect endogenous expression of the examined retinoid-augmented species.

### Changes in lipid droplet size or ATGL levels do not affect G0S2 inhibitory effects

**Figure 5:**
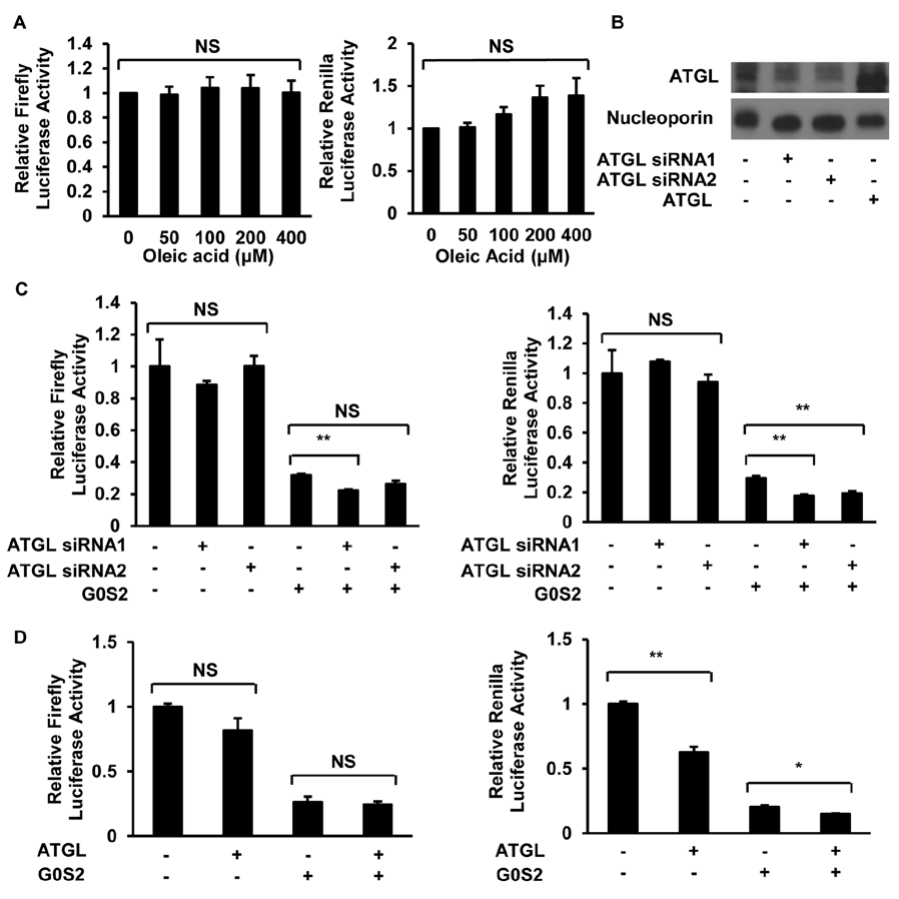
Changes in lipid droplet size or ATGL level do not appreciably affect G0S2 inhibitory function. (A) Oleic acid treatment of ED-1 cells to increase lipid droplet size did not affect firefly (left panel) or renilla (right panel) luciferase activity. Results were averaged from four independent studies. (B) Immunoblot assays confirmed that the ATGL siRNAs and ATGL overexpression vector functioned as expected in A549 cells. Nucleoporin expression served as a loading control. (C) ATGL knockdown by siRNA did not reproduce the inhibitory effects of G0S2 on firefly (left panel) or renilla (right panel) activities in A549 cells. Results were averaged from three independent experiments. (D) Engineered ATGL overexpression did not reverse the inhibitory effect of G0S2 on firefly (left panel) or renilla (right panel) luciferase activities in A549 cells. Results were from three independent experiments. Error bars represent standard errors. NS indicates a comparison that is not significant. ^*^P<0.05 and ^**^P<0.01 represent statistical significance.

G0S2 interacts with and inhibits ATGL and this can decrease lipolysis and increase cellular lipid droplet size (13). Since G0S2 interacts with ATGL via its HD domain and because the HD domain conferred G0S2 inhibitory effects, the role of ATGL-regulated lipolysis in this repression was examined. ED-1 cells were treated with oleic acid to increase lipid droplet size (13). This experiment was designed to investigate whether mimicking inhibition of lipolysis reproduced G0S2 repressive effects. Oleic acid treatment of ED-1 cells markedly increased lipid droplet size even at the lowest concentration tested (50 *μ*M), as visualized by BODIPY staining (data not shown). Yet, the reporter activity of the indicated transiently transfected luciferase constructs did not exhibit inhibition after oleic acid treatment (Fig. 5A).

To learn whether ATGL plays a role in the suppressive effect of G0S2, ATGL levels were reduced by siRNA knockdown (Fig. 5B and C) and independently increased by gain of ATGL expression (Fig. 5D). G0S2 inhibitory effects were minimally affected by ATGL knockdown or gain of ATGL expression as shown in Fig. 5C and D. Luciferase reporter constructs were transiently co-transfected into A549 cells (in the presence or absence of G0S2 transfection) with individual ATGL-targeting versus control siRNAs. In A549 cells that overexpressed the empty vector pCMV-myc, co-transfection of individual ATGL-targeting siRNAs did not appreciably decrease the respective luciferase activity as compared to cells co-transfected with control siRNA (Fig. 5C). In A549 cells overexpressing G0S2, there was a minor decline in luciferase activity between ATGL siRNA- and control siRNA-co-transfected cells, but this was not as large as the inhibitory effect caused by the transfection of G0S2 alone versus its empty vector control. In addition, co-transfection of ATGL and G0S2 in A549 cells did not reverse the inhibitory effect of G0S2 on luciferase activity (Fig. 5D). This argued against ATGL playing a driving role in the observed G0S2 repression.

### G0S2 localization

**Figure 6:**
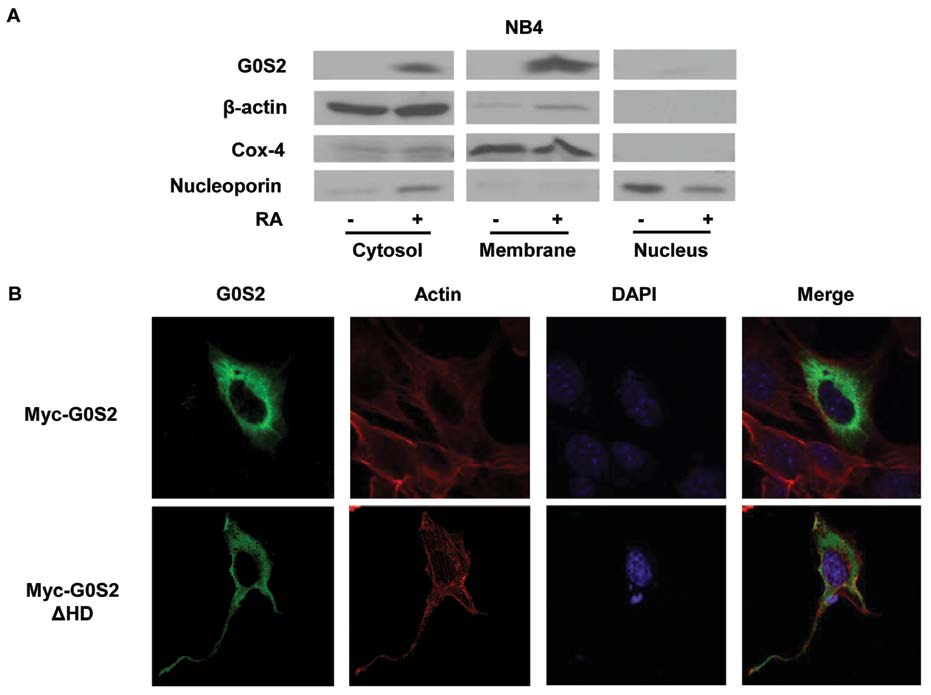
G0S2 subcellular localization and the importance of the HD domain. (A) NB4 APL cells were treated with RA (1 *μ*M) to augment endogenous G0S2 expression and these cells underwent subcellular fractionations to isolate cytosolic, membrane and nuclear compartments. Immunoblot analyses showed that G0S2 was prominently detected in the cytosol and membrane, but not in the nucleus. Actin, cox-4 and nucleoporin served as loading controls for the cytosolic, membrane and nuclear compartments, respectively. (B) ED-1 cells were transfected with myc-tagged wild-type G0S2 (upper panel) or with myc-tagged G0S2 ΔHD (lower panel) and imaged for G0S2 (with an anti-myc antibody), actin (with phalloidin to visualize microfilaments) or DNA (with DAPI). Cells were visualized by confocal microscopy. Wild-type G0S2 (myc-G0S2) was distributed throughout the cytoplasm and concentrated in the perinuclear region. In contrast, myc-G0S2 ΔHD was distributed more diffusely throughout the cytoplasm, especially in the cell processes and projections.

Subcellular localization of G0S2 was next examined. To investigate the subcellular localization of G0S2 protein, NB4 cells were treated with RA (1 *μ*M) for 2 days to induce endogenous G0S2 expression. In the absence of RA-treatment G0S2 was not appreciably expressed. Subcellular fractionation and immunoblot analysis after RA-treatment of NB4 cells revealed that endogenous G0S2 was predominantly expressed in the cytosolic and membrane fractions of these cells (Fig. 6A). This subcellular localization of G0S2 was independently determined in ED-1 cells, where ED-1 cells were transfected with myc-G0S2 and probed with an anti-myc antibody to detect G0S2 protein, and also with Phalloidin to detect microfilaments. As visualized by confocal microscopy, G0S2 was distributed in the cytoplasm, especially in the peri-nuclear region, but with relatively diffuse cytoplasmic staining (Fig. 6B). Interestingly, after its transfection into ED-1 cells, the myc-G0S2 ΔHD protein was distributed diffusely throughout the cytoplasm and G0S2 expression was extended to cellular processes and projections, with reduced perinuclear staining as compared to transfected wild-type G0S2 (Fig. 6B).

## Discussion

G0S2 is an RA target gene (9) and its functions are under active study. Studies performed here in APL cells found that G0S2 was induced after treatment with RAR, but not RXR agonists (Fig. 1). It is previously reported that G0S2, via its HD domain, complexes with ATGL and inhibits ATGL lipolytic activity (13). The current study advanced prior work by identifying a previously unrecognized G0S2 function. This is its ability to inhibit exogenous reporter activities, as shown in Figs. 1, 2 and 3. Yet, G0S2 did not appear to affect endogenous gene expression, as displayed in Fig. 4. G0S2 repressed transcriptional activities of several retinoid responsive reporter plasmids including those containing individual regulatory elements for RARβ, UBE1L or G0S2 itself; this occurred in diverse cell lines including those that were retinoid responsive or not, as found in Figs. 1, 2 and 3. This inhibitory effect was antagonized by siRNA-mediated knockdown of G0S2 (Fig. 1). The inhibitory effects of G0S2 were not restricted to retinoid responsive reporter plasmid activities. Both firefly luciferase and renilla luciferase activities as well as GFP fluorescent intensities driven by constitutively active promoters had decreased activity or expression in the presence of G0S2 co-transfection (Fig. 3 and data not shown). Notably, this decline could not be explained by a substantial change in transfection efficiency as noted in Fig. 1 (and data not shown). Stable G0S2 expression also inhibited exogenous reporter activities (Fig. 3), which indicated that these inhibitory effects depended on expression of G0S2 protein.

Interestingly, neither transient nor stable expression of G0S2 (Fig. 4) affected endogenous levels of retinoid regulated species. This indicated that the G0S2 inhibitory effect appears restricted to the regulation of exogenous reporter activities. The deletion of the G0S2 HD domain at least partially rescued G0S2 inhibitory effects, indicating that this domain played a direct role in conferring G0S2-mediated transcriptional repression (Fig. 3). Yet, this activity was likely independent of the ATGL inhibitory function of G0S2 because respective engineered gain or loss of ATGL expression as well as induced changes in lipid droplet size after oleic acid treatment each did not appreciably affect reporter construct activities (Fig. 5). G0S2 exerted these actions via its predominant localization to the cytosol and membrane rather than to the nuclear compartment, as established in Fig. 6. Yet, deletion of the G0S2 HD domain altered both the repressive effects of G0S2 and its subcellular localization, as shown in Figs. 3 and 6. In contrast to the localization of wild-type G0S2, the ΔHD mutation of G0S2 was localized to the cytoplasm in a more prominent pattern within cellular processes and projections (Fig. 6). This could contribute to the reduced transcriptional repression exerted by this G0S2 mutation.

A repressive effect on exogenously expressed species, but not on endogenous gene expression was previously reported. It was found that spermidine/spermine N1-acetyltransferase 1 (SSAT1) exerted similar effects as reported here for G0S2 (28). SSAT1 is an enzyme involved in polyamine catabolism and it catalyzes the N1-acetylation of spermidine and spermine to form acetyl derivatives (28). Like G0S2, SSAT1 inhibited expression of exogenously expressed proteins including GFP and GFP-elF5A (28). The precise mechanism responsible for this inhibition by SSAT1 was not found (28). Yet, similar to that described here for G0S2, the repressive effect of SSAT1 was also independent of increased protein degradation since proteasome, protease, lysosome or autophagy inhibitors did not antagonize SSAT1 repressive activity (28).

SSAT1-dependent repression was limited to transiently transfected SSAT1 species (28). Neither the induction of endogenous SSAT1 by a potent SSAT1 inducer such as BENSpm nor stable expression of SSAT1 repressed exogenously examined proteins (28). These data indicated that the inhibitory effects of SSAT1 were likely due to its transient transfection. In contrast to SSAT1, stable expression of G0S2 also repressed activities of exogenously expressed reporter plasmids as shown in Fig. 3B, implicating a direct role for G0S2 protein in this observed inhibition. Other factors could still play a role in this process since the extent of inhibition by stable G0S2 protein expression was less than observed after transient G0S2 transfection. Even so, changes in transfection efficiency conferred by introduction of a G0S2 expression vector played at most a minor role in this effect, as displayed in Fig. 1C (and in data not shown).

G0S2 was previously reported to complex with and inhibit ATGL, which reduced lipolysis and increased lipid droplet size in cells (13). Because the G0S2 HD domain was responsible for this interaction with ATGL and was the domain that antagonized the repressive effects of G0S2 described in this study, it was hypothesized that transcriptional repression by G0S2 was also mediated by ATGL. Intriguingly, this was not found to be the case since mimicking the consequences of increased G0S2 expression by increasing lipid droplet size via oleic acid treatment did not reproduce this G0S2 inhibition (Fig. 5). In addition, ATGL overexpression did not reverse G0S2 repression (Fig. 5). Consistent with this observation, the siRNA-mediated knockdown of ATGL did not mimic G0S2 repressive effects (Fig. 5). Hence, ATGL-regulation of lipolysis does not likely play a major role in this G0S2 inhibitory effect. When ATGL targeting siRNA was transfected with a G0S2 overexpression vector, it slightly increased the G0S2 repressive effect, but the degree of this inhibition was not as great as observed with G0S2 alone (Fig. 5C). This could be due to a reduction of the G0S2-ATGL complex after ATGL knockdown, leading to the release of free G0S2 that can then exert an inhibitory effect.

It was previously reported that G0S2 was localized to the endoplasmic reticulum (ER) and this conclusion was based on the subcellular localization of GFP-tagged G0S2 (12). The subcellular localization findings presented here likely diverge from this prior work because the GFP-tagged G0S2 used was much larger than the native G0S2 protein. This GFP-tag could lead to non-physiologic localization of G0S2. Consistent with this interpretation was the observation that introduction of GFP-tagged G0S2 in studied cells did not repress reporter activities to the same extent as myc-tagged G0S2 (data not shown). Unlike GFP-tagged G0S2, myc-tagging leads to a slight change in the size of G0S2 protein and this did not appear to affect subcellular localization versus endogenously induced G0S2 protein, as shown in Fig. 6. In the current study, a prominent localization of G0S2 to the ER compartment was not appreciated.

The biological basis for the inhibitory effect of G0S2 is not well understood. Yet, there are several plausible explanations. G0S2 expression is enhanced in autoimmune and inflammatory diseases (15–17). Perhaps the G0S2 inhibitory effect is related to a G0S2 role in immunity. A class of small proteins known as antimicrobial peptides (AMPs) is implicated in innate immunity (29). LL-37, one of the most studied AMPs, has an ability to bind DNA (30). It is conceivable that G0S2 exerts its inhibitory effect on the diverse reporter plasmids shown in the studies presented here through this recognized function of antimicrobial peptides. Future study will explore this possibility. If this provides a mechanistic basis for the observed inhibitory actions of G0S2, this finding would establish a previously unrecognized role for G0S2 beyond what has recently been found (31).

Of course, to discern a potential role for G0S2 in immunity, it is necessary to explore the *in vivo* activities of G0S2. In this regard, it is interesting that G0S2 transgenic mice exist (17). Although they did not exhibit an obvious immune system abnormality, these mice did have evidence for this because increased autoimmunity-related antibodies were detected in their serum as compared to wild-type mice (17). To build on this prior study, it would be useful to engineer a G0S2 knockout mouse model in the future. This would be a new tool to discern the precise biological role for G0S2 beyond its known role in regulating metabolism (31). Until such a model is at hand, our data extend the prior study by highlighting a previously unrecognized G0S2 activity. This is the ability of the retinoic acid target gene G0S2 to repress both exogenous gene expression and reporter activity.
